# Supplementary figures and images for: Ribosomal RNA synthesis by RNA polymerase I is subject to premature termination of transcription
Source: eLife. 2026 Feb 12;14:RP106503. doi: 10.7554/eLife.106503 (PMC12900514; doi:10.7554/eLife.106503)

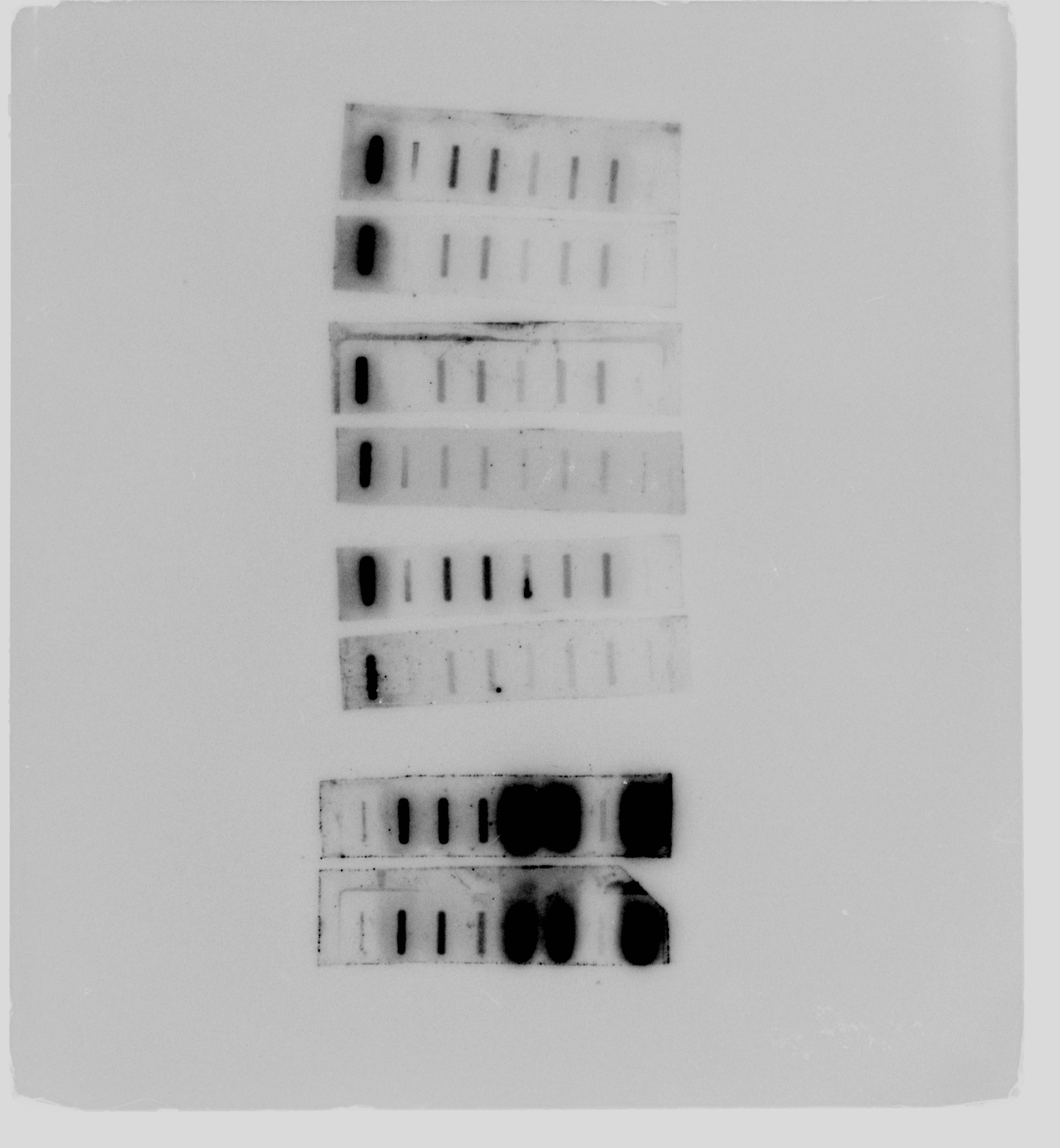

Supplement: Figure 1—source data 1. [file elife-106503-fig1-data1.zip › Figure 1-source data 1.tif]

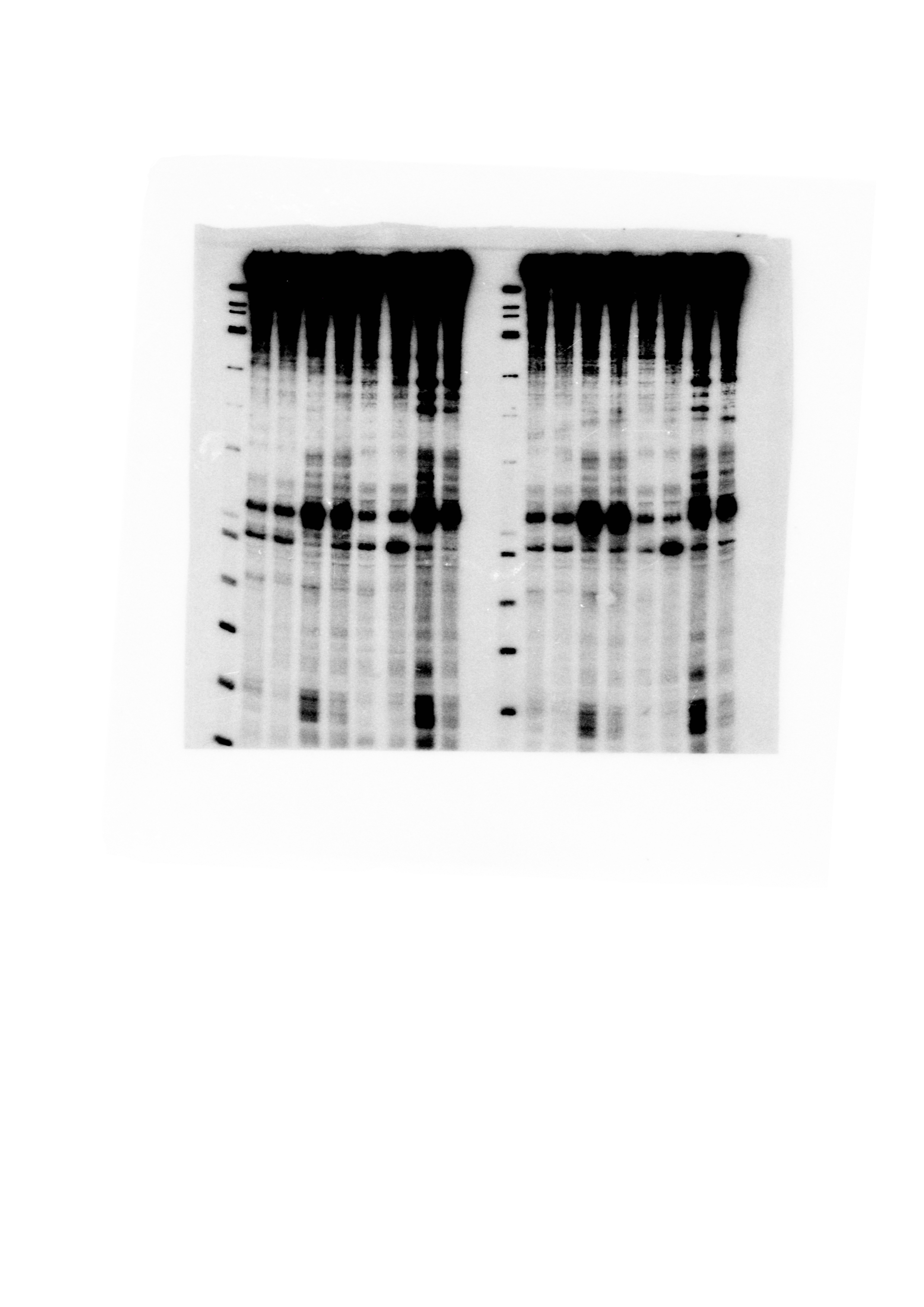

Supplement: Figure 3—source data 1. [file elife-106503-fig3-data1.zip › Figure 3B-source data 1 A.tiff]

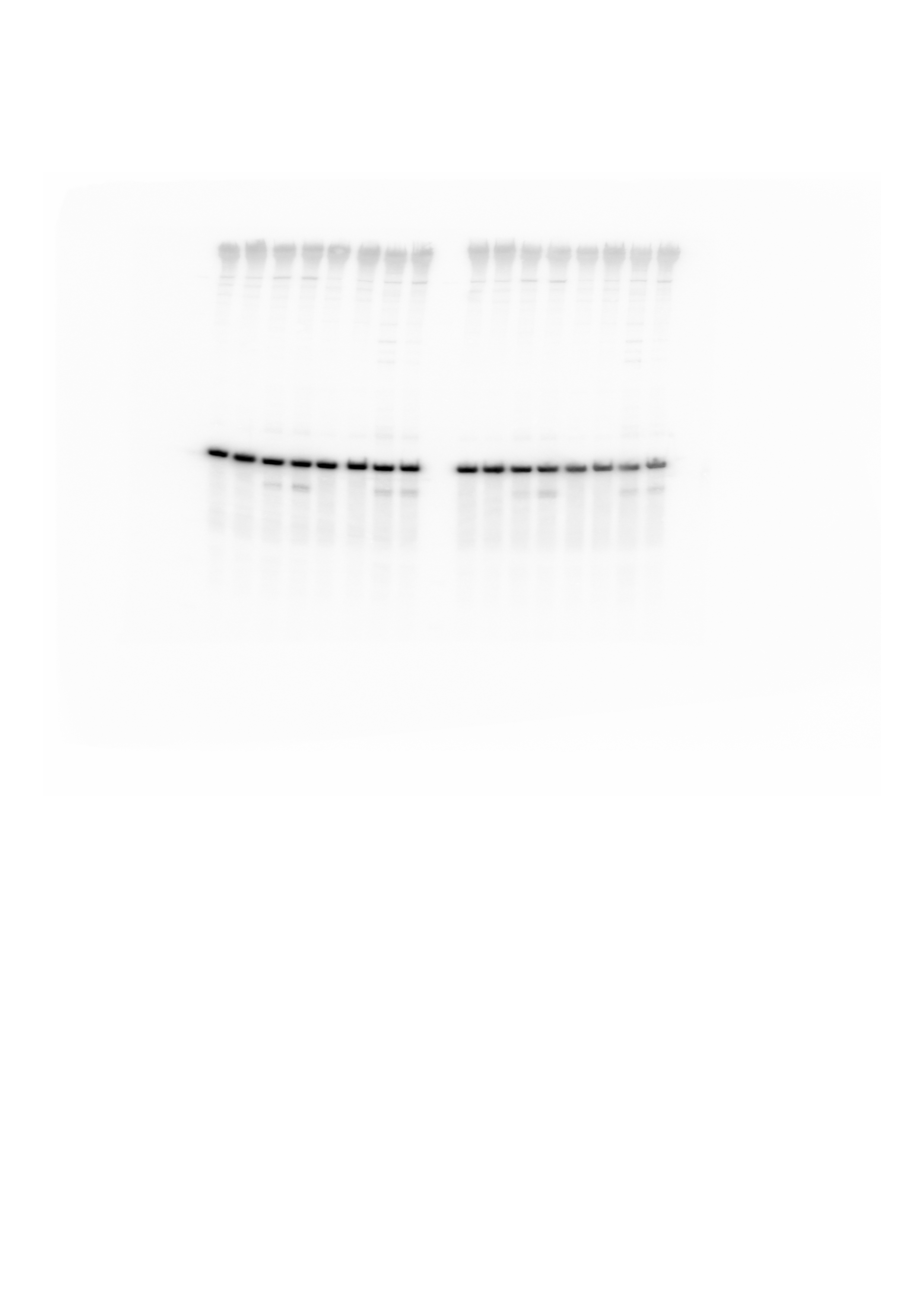

Supplement: Figure 3—source data 1. [file elife-106503-fig3-data1.zip › Figure 3B-source data 1 B.tiff]

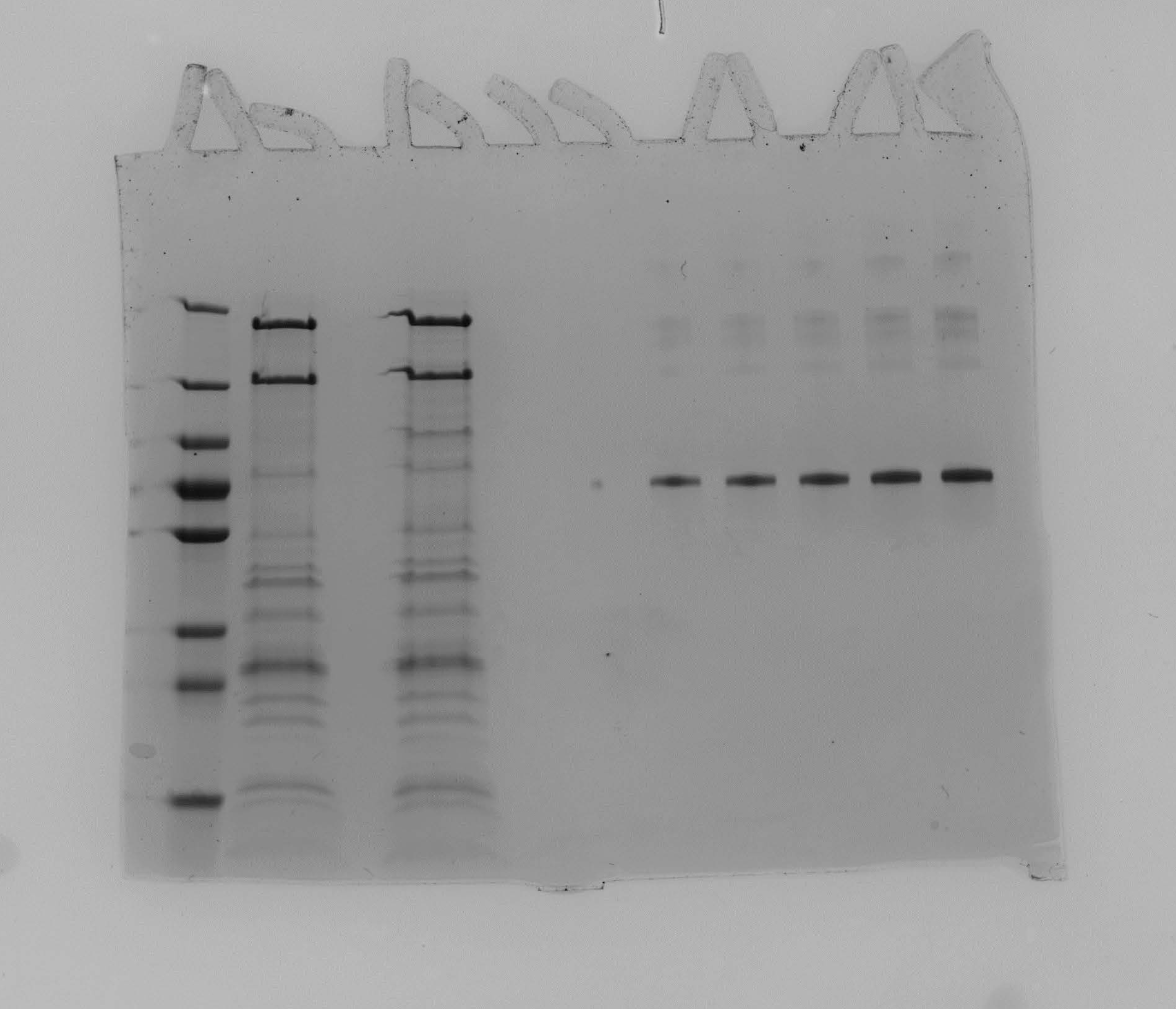

Supplement: Figure 3—source data 2. [file elife-106503-fig3-data2.zip › 4A.tiff]

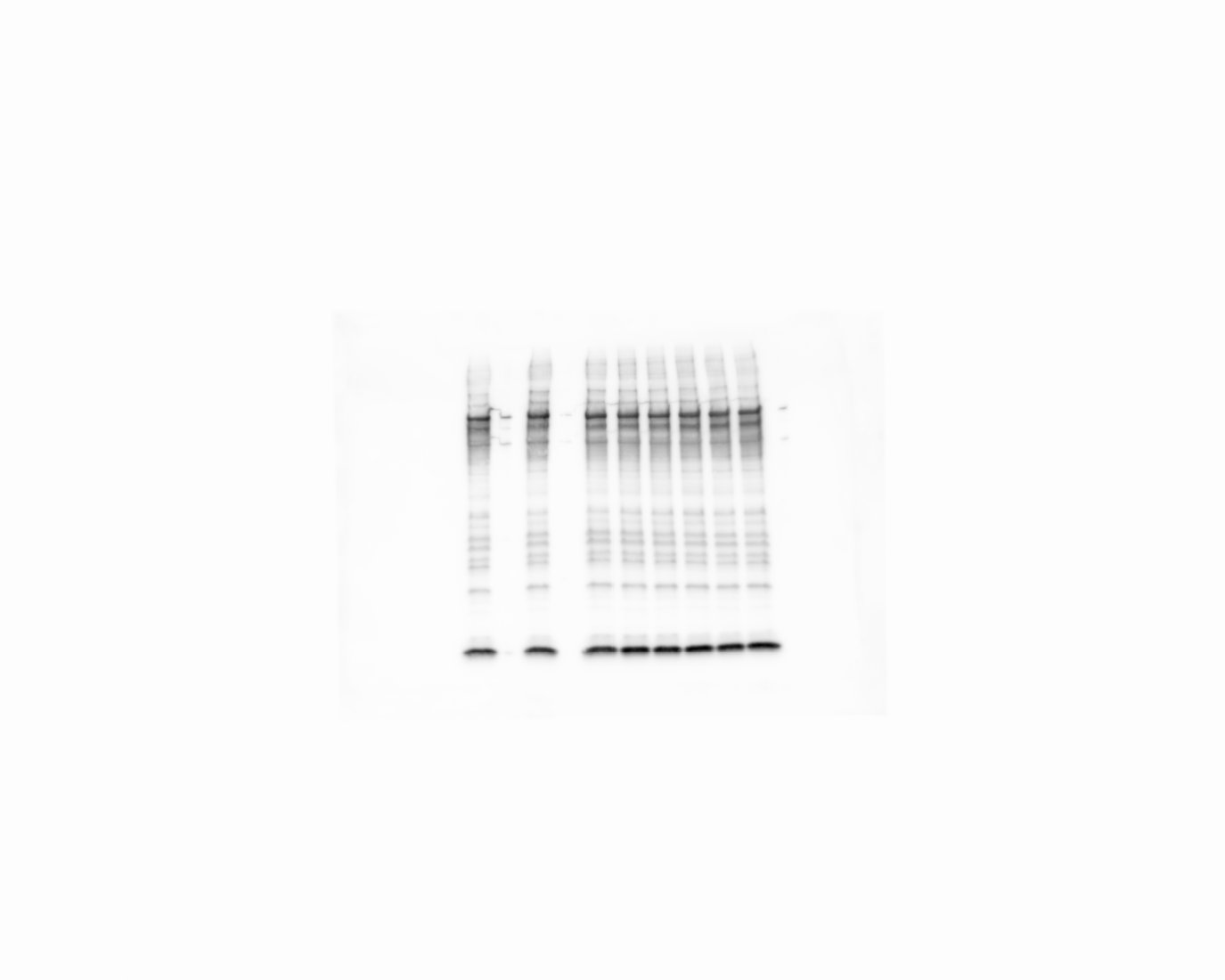

Supplement: Figure 3—source data 2. [file elife-106503-fig3-data2.zip › 4B.tif]

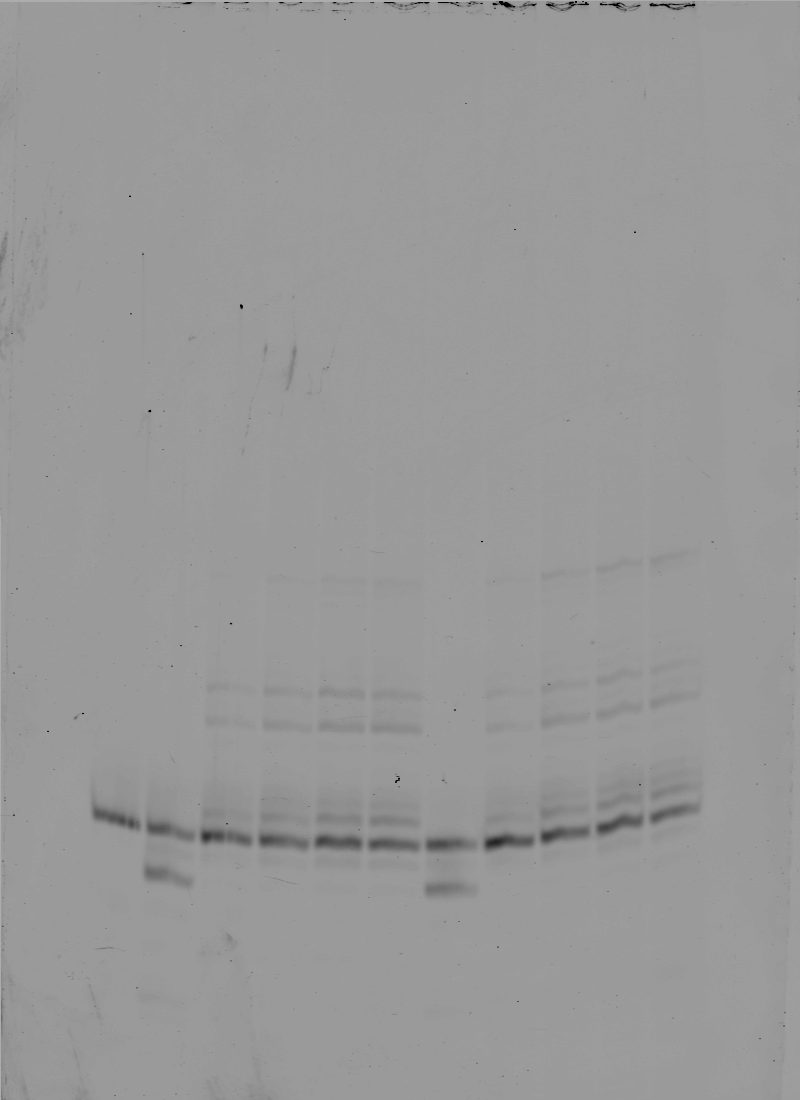

Supplement: Figure 4—figure supplement 1—source data 2. [file elife-106503-fig4-figsupp1-data2.zip › Figure 4 sup1 E.tif]

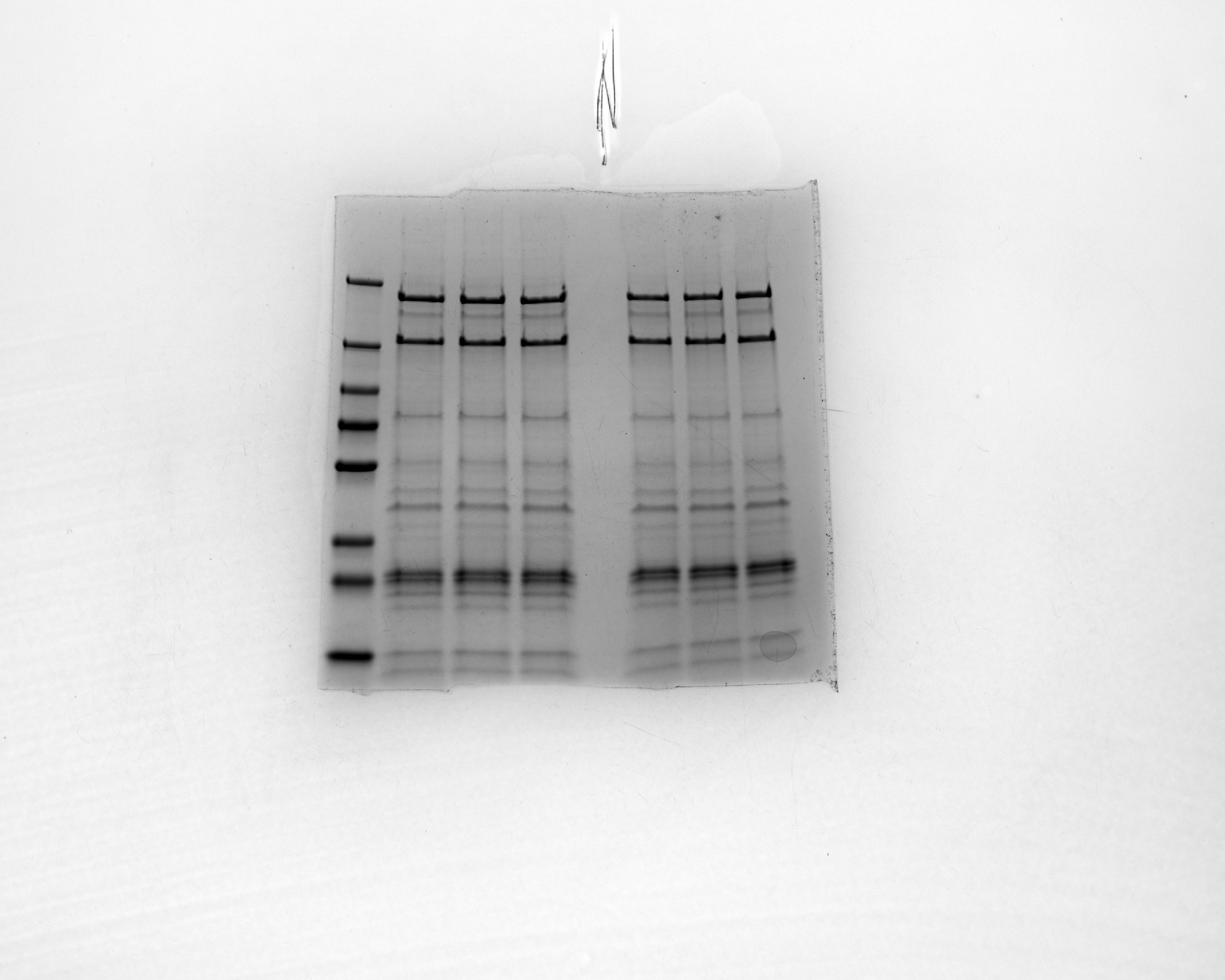

Supplement: Figure 4—figure supplement 1—source data 2. [file elife-106503-fig4-figsupp1-data2.zip › Figure 4 sup 1 A.tif]

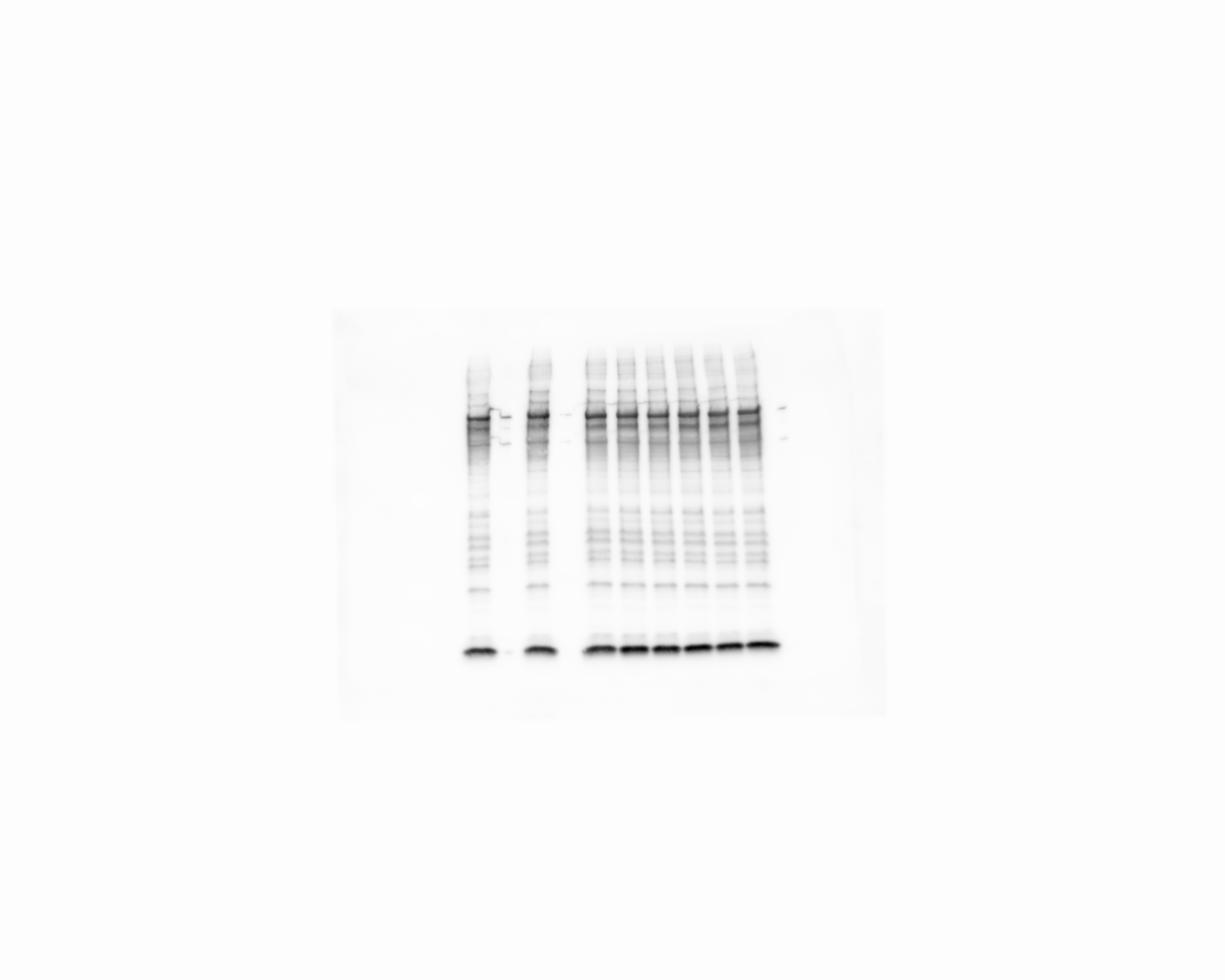

Supplement: Figure 4—figure supplement 1—source data 2. [file elife-106503-fig4-figsupp1-data2.zip › Figure 4 sup1 B.tif]

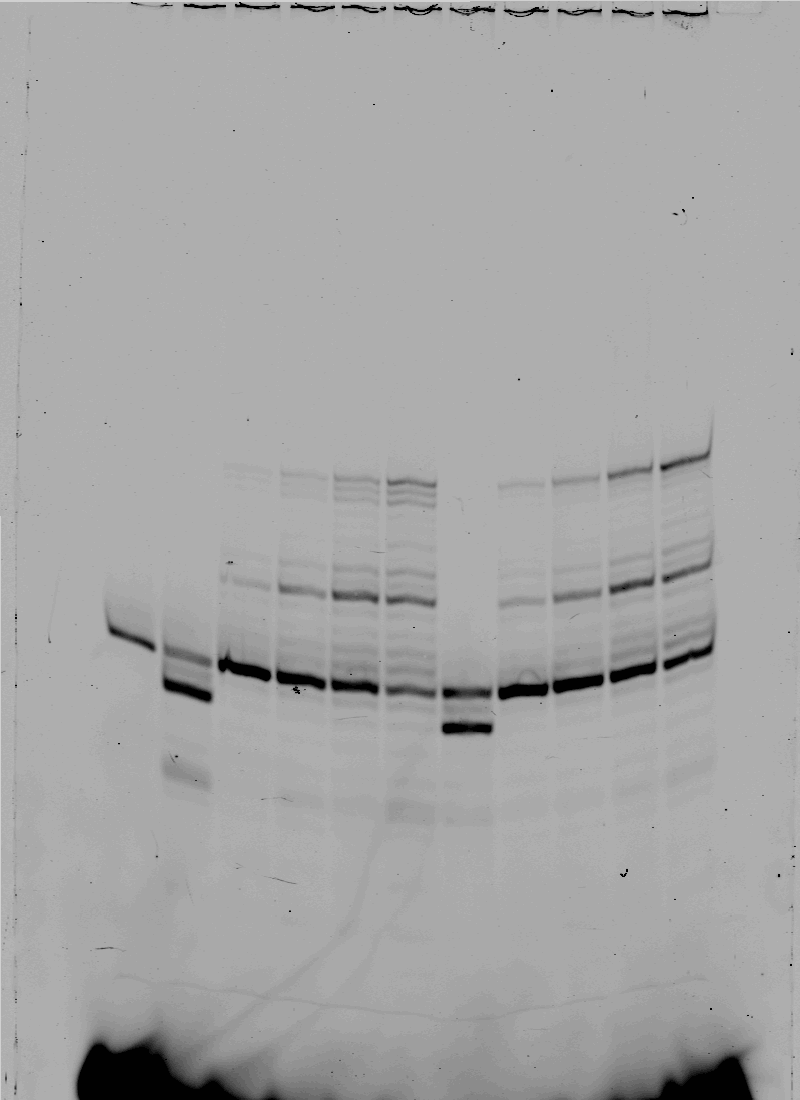

Supplement: Figure 4—figure supplement 1—source data 2. [file elife-106503-fig4-figsupp1-data2.zip › Figure 4 sup1 C.tif]

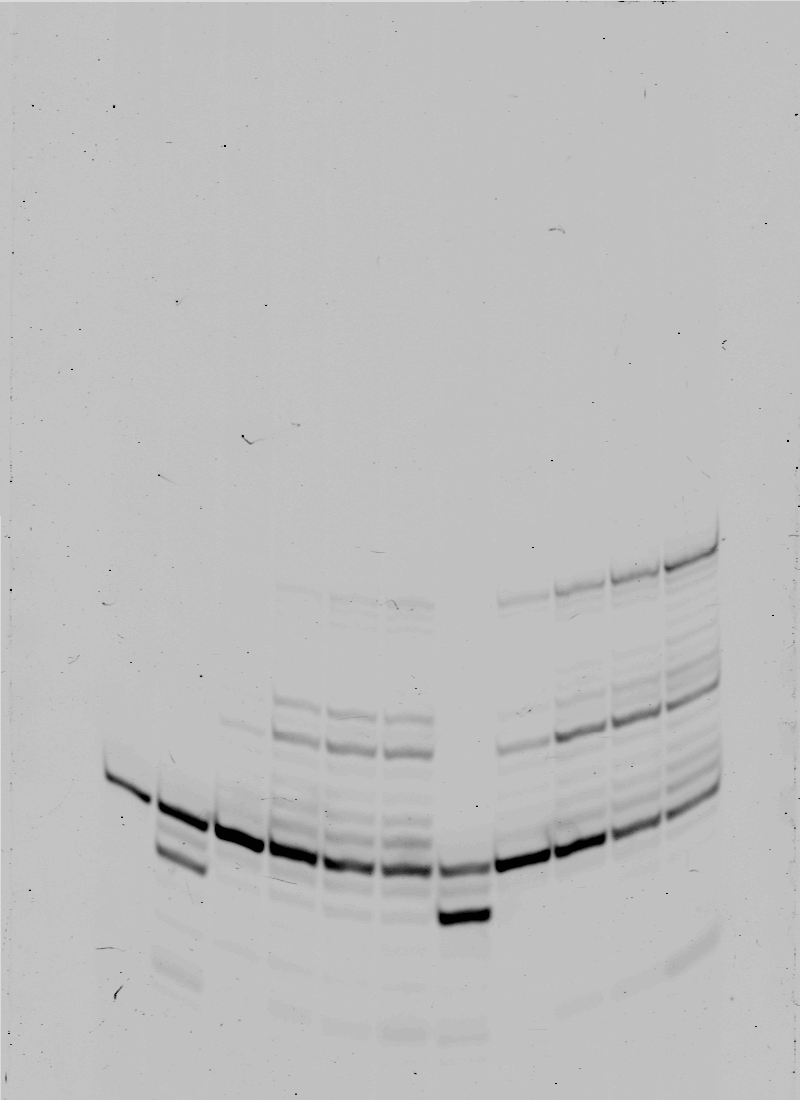

Supplement: Figure 4—figure supplement 1—source data 2. [file elife-106503-fig4-figsupp1-data2.zip › Figure 4 sup1 D.tif]

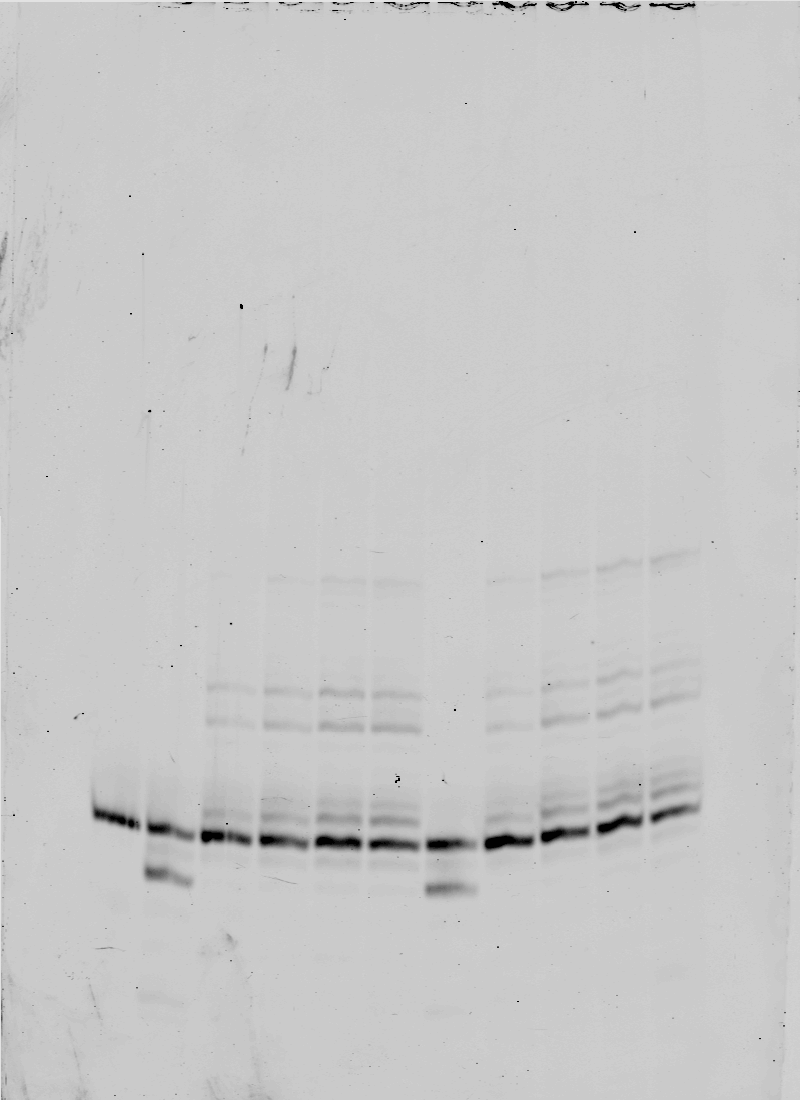

Supplement: Figure 5—source data 1. [file elife-106503-fig5-data1.zip › Figure 5E.tif]

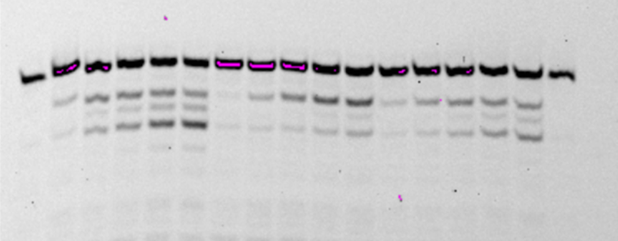

Supplement: Figure 5—source data 1. [file elife-106503-fig5-data1.zip › 5B.tiff]

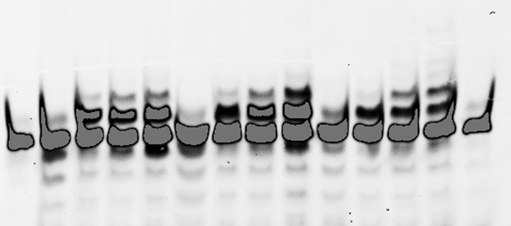

Supplement: Figure 5—source data 1. [file elife-106503-fig5-data1.zip › 5H.tiff]

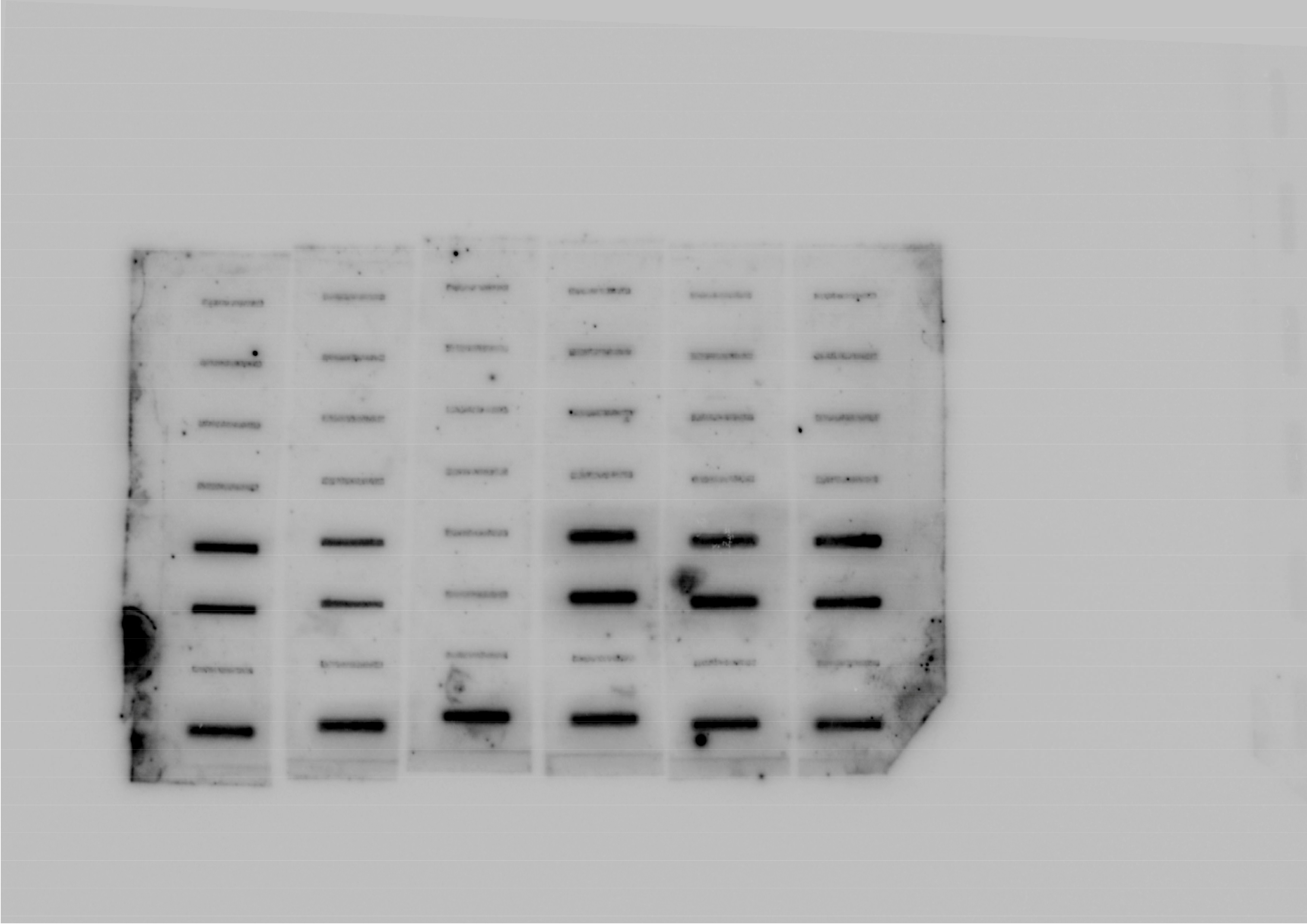

Supplement: Figure 6—source data 2. [file elife-106503-fig6-data2.zip › Figure 6B.tiff]

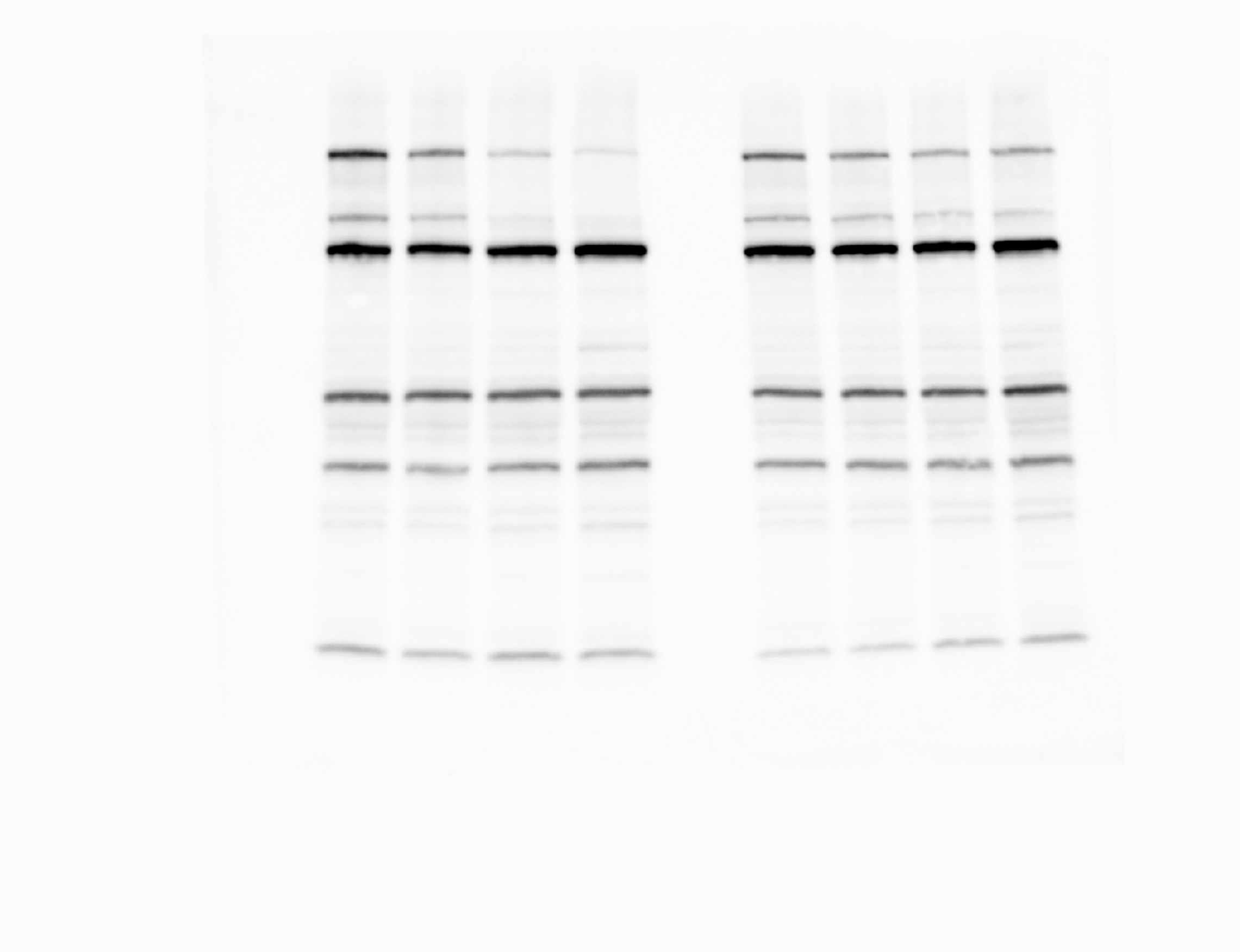

Supplement: Figure 6—source data 2. [file elife-106503-fig6-data2.zip › Figure 6C.tif]

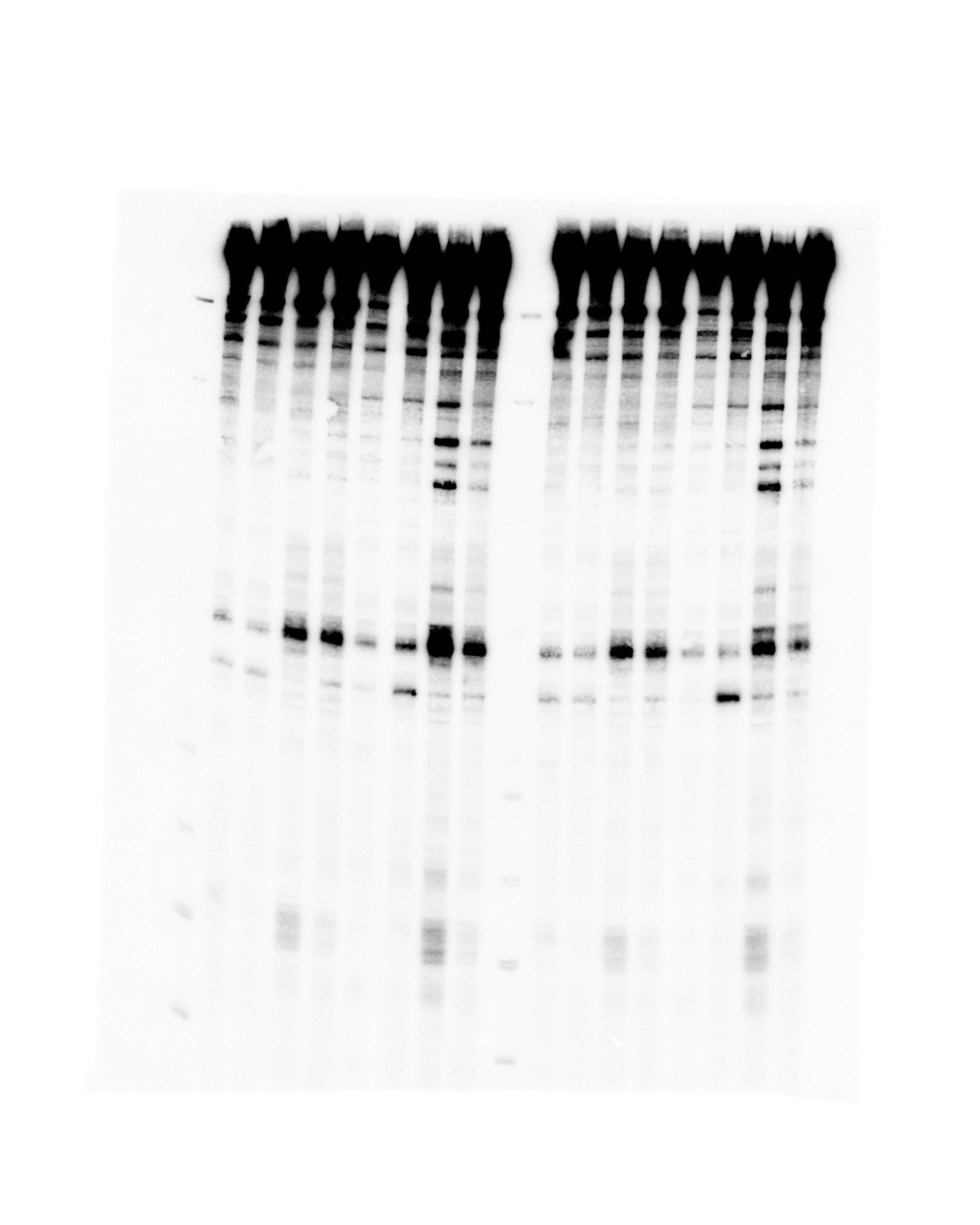

Supplement: Figure 7—source data 2. [file elife-106503-fig7-data2.zip › Figure 7C-source data 1 A.tiff]
